# Antipyrin and Sweet Spirits of Nitre

**Published:** 1889-03

**Authors:** John A. Miller

**Affiliations:** Buffalo, N. Y., Lecturer on Medical Chemistry and Toxicology, Medical Department, Niagara University; Chemical Laboratory of the Niagara University


### *ANTIPYRIN AND SWEET SPIRITS OF NITRE.*

LABORATORY NOTE.

By JOHN A. MILLER, M. Sc., Ph. D., Buffalo, N. Y.,

Lecturer on Medical Chemistry and Toxicology, Medical Department, Niagara University.

Physicians and pharmacists who have endeavored to mix antipyrin and sweet spirits of nitre, have doubtless observed the beautiful green-colored solution obtained. If this solution be allowed to stand, beautiful green-colored crystals are deposited.

What are these crystals? And to what is their formation due?

Further than that these crystals are poisonous, the medical and pharmaceutical journals say but little, if anything, of the cause of their formation.

The ordinary commercial sweet spirits of nitre consists essentially of an alcoholic medium, containing from two to five per cent. of ethyl nitrite in solution. Small quantities of aldehyde, and nitrous and acetic acids are always present.

To which of these compounds is the formation of this green color due?

In order to ascertain whether this coloration was due to the ethyl nitrite and aldehyde, I neutralized the free acids contained in a sample of sweet spirits of nitre, and found that upon mixing the two solutions they remained perfectly colorless, even after the expiration of forty-eight hours. This proved that the incompatibility of these two substances was due to one, or both, of the free acids present in the original solution of nitrous ether.

As antipyrin is nothing more than the hydrochloric acid salt of the basic compound, dimethyloxychinizin ($C_{11}$, $H_{12}$, $N_2$ O), it did not seem rational that this change was caused by the presence of free acetic acid. I, therefore, investigated the action of nitrous acid upon antipyrin. Upon passing nitrous acid anhydride into an aqueous solution of antipyrin, the solution first became dark green and finally brown; a brown flocculent precipitate separating at the same time. Believing that the action had been carried too far, I added an aqueous solution of nitrous acid to a solution of antipyrin, and obtained a beautiful green-colored solution, which deposited green-colored crystals upon standing. The addition of a large excess of nitrous acid transformed the green solution to a brown one, as previously stated.

The members of the medical profession will now be able to combine these two remedial agents, knowing that the pharmacist, by a neutralization of the free acids contained in sweet spirits of nitre, can compound his prescription without any danger of a poisonous substance being formed. Having seen, some little time since, a prescription which called for antipyrin, sweet spirits of nitre and *aromatic sulphuric acid*, I cannot omit mentioning that any free acid is incompatible with the two first-named compounds. The presence of a free acid would cause the liberation of the nitrous acid from its combination with soda or potash, thus leaving it free to act upon the antipyrin present.

The chemical composition and physiological action of this green compound is at present being investigated.

Chemical Laboratory of the Niagara University.
Buffalo, Jan. 16, 1889.

